# Multiple rib fractures confused with loss of lung volume on chest X‐ray

**DOI:** 10.1002/ccr3.6179

**Published:** 2022-08-14

**Authors:** Risa Hirata, Masaki Tago, Motoko Tago

**Affiliations:** ^1^ Department of General Medicine Saga University Hospital Saga Japan; ^2^ Department of Cardiovascular Medicine Saga University Saga Japan

**Keywords:** chest X‐ray, multiple rib fractures, older people

## Abstract

A 90‐year‐old woman with osteoporosis and no recent injury history visited the hospital for a regular checkup. Chest X‐ray showed a loss of right upper lung volume. Although we suspected pulmonary parenchymal or pleural disease, computed tomography revealed multiple rib fractures on the right side, which caused thoracic deformation.

## Abstract 2

Multiple rib fractures without a history of injury can present with similar findings to severe trauma on chest X‐ray. Physicians should carefully check chest X‐rays because findings of a thoracic deformity can be confused with pleural diseases.

## 1 CASE IMAGE

**FIGURE 1:**
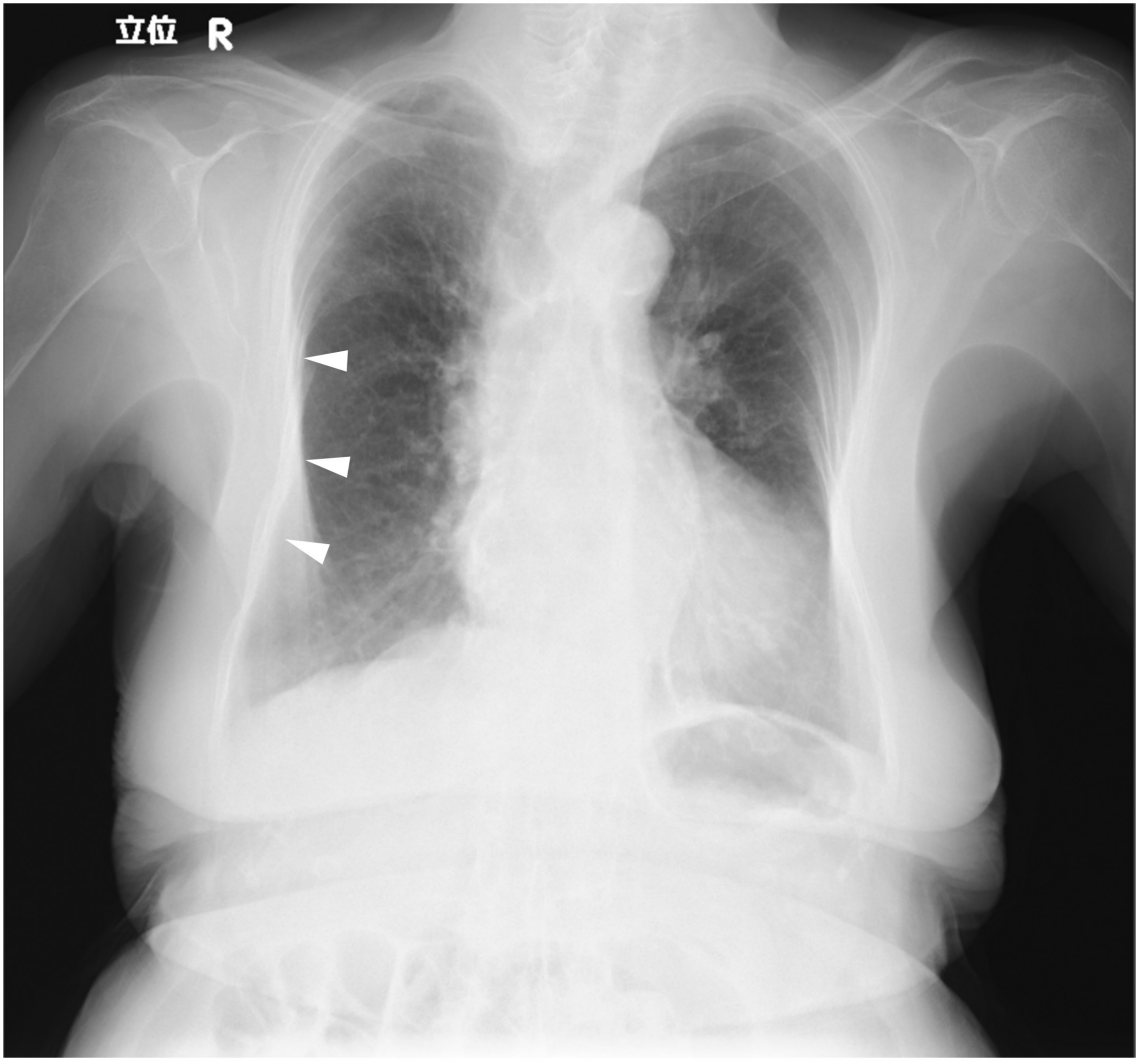
Chest X‐ray 10 months prior. Chest X‐ray shows a slight deformity of the right thorax (arrowheads), and no abnormality in the lung field.

**FIGURE 2:**
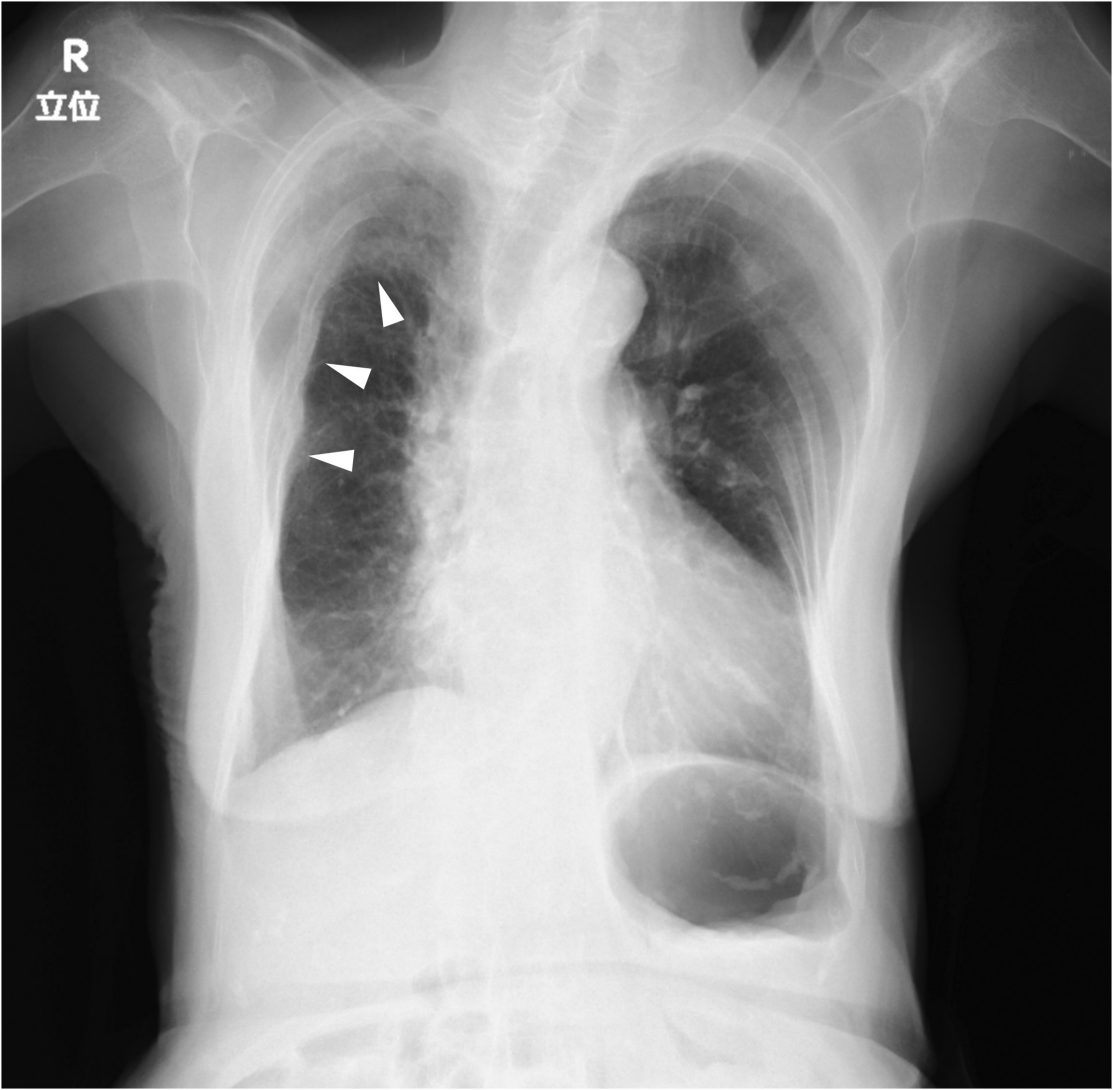
Chest X‐ray obtained at the present visit. Chest X‐ray shows more severe deformation of the right thorax and a low‐density area on the right upper lung field (arrowheads).

**FIGURE 3:**
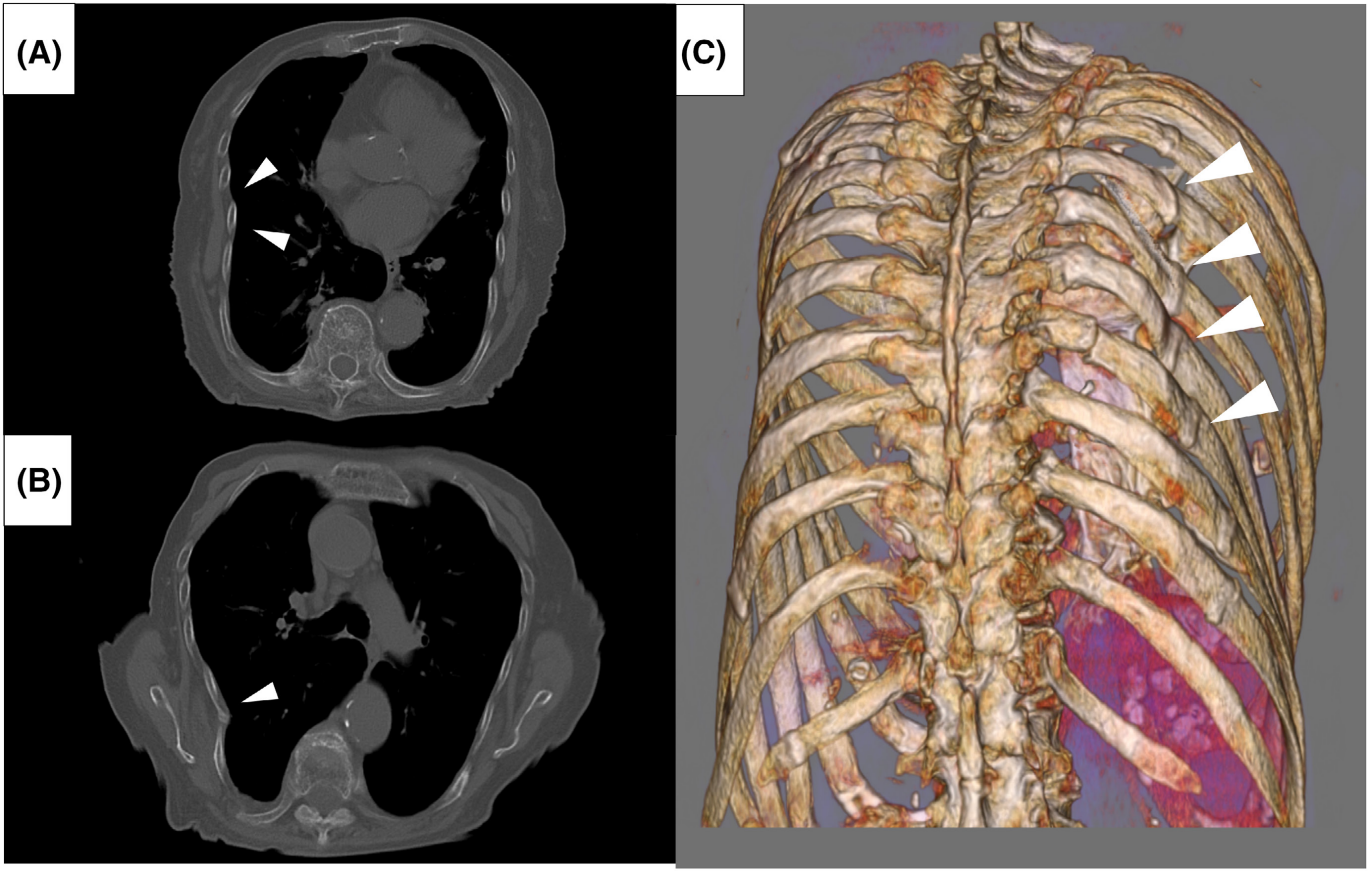
Chest computed tomography without contrast enhancement. The axial images of the bone window show multiple rib fractures with callus formation (A, B: arrowheads). The reconstructed image clearly shows multiple rib fractures with thoracic deformation (C: arrowheads).

A 91‐year‐old woman with osteoporosis visited the hospital for a regular checkup. She had a history of a 5th rib fracture and multiple vertebral compression fractures. However, a chest X‐ray taken 10 months prior showed no findings in the pulmonary field (Figure 1). She had no complaints, recent injury history, or abnormal physical findings. Chest X‐ray at admission incidentally revealed loss of right upper lung volume (Figure 2). Although we suspected pulmonary or pleural disease, computed tomography only revealed multiple fractures with callus formation from the 4th to 12th right ribs and thoracic deformation (Figure 3). The patient was diagnosed with a thoracic deformity caused by rib fractures and was followed up because she had no complications.

The patient's chest X‐ray was similar to a stove‐in‐chest image, in which the flail segment is sunken because of severe trauma.^1^ Older patients can exhibit multiple rib fractures from minor trauma, even if they have no injury history. Additionally, thoracic deformation or thickening of the surrounding soft tissue can be mistaken for pulmonary parenchymal or pleural lesions based on chest X‐ray findings.^2^ Therefore, physicians should carefully check the ribs and thoracic shape on chest X‐ray before performing computed tomography.

## AUTHOR CONTRIBUTIONS

RH was involved in the literature search, study conception, and drafting of the manuscript. Masaki T was involved in the literature search, study conception, and drafting and revision of the manuscript. Motoko T was involved in the literature search, drafting of the manuscript, and clinical care of the patient.

## CONFLICT OF INTEREST

The authors state that they have no conflict of interest.

## ETHICAL APPROVAL

This manuscript conforms to the provisions of the Declaration of Helsinki in 1995 (as revised in Brazil 2013).

## CONSENT

Written informed consent was obtained from the patient to publish this report in accordance with the journal’s patient consent policy.

## Data Availability

The data that support the findings of this study are available from the corresponding author upon reasonable request.
